# Forest Bathing (*Shinrin-yoku*) and Preventive Medicine: Immune Modulation, Stress Regulation, Neurocognitive Resilience, and Neurological Health

**DOI:** 10.3390/medsci14010095

**Published:** 2026-02-15

**Authors:** Arnab Bandyopadhyay, Soumya Shah, Giovanni N. Roviello

**Affiliations:** 1 Department of Molecular Medicine and Medical Biotechnology, University of Naples Federico II, Via Tommaso De Amicis 95, 80131 Naples, Italy; 2 Institute of Biostructures and Bioimaging, Italian National Research Council (IBB-CNR), Via P. Castellino 111, 80131 Naples, Italy

**Keywords:** forest bathing, preventive medicine, immune modulation, neurocognitive resilience, stress physiology, neuroprotection, Parkinson’s disease, stroke recovery

## Abstract

**Background/Objectives:** Forest bathing (*Shinrin-yoku*) is a nature-based approach with potential preventive health relevance. This review summarizes evidence on its effects on immune function, stress physiology, and neuroprotective pathways. **Methods:** A narrative review of peer-reviewed studies was conducted using major scientific databases, including observational and interventional research assessing physiological or neurocognitive outcomes following forest exposure. **Results:** Forest bathing is associated with enhanced natural killer (NK) cell activity, modulation of inflammatory cytokine profiles, reductions in cortisol levels, and shifts toward parasympathetic autonomic dominance. Evidence also suggests a contributory role of tree-derived biogenic volatile organic compounds and phytoncides in immune and stress-regulatory effects. Emerging findings indicate potential benefits for cognitive restoration, emotional regulation, and neurotrophic signaling; however, substantial heterogeneity in study design, exposure characteristics, and outcome measures limits direct comparability and causal inference. **Conclusions:** Current evidence supports forest bathing as a promising, low-risk strategy for supporting immune resilience, stress regulation, and neurocognitive well-being within a preventive health framework. Preliminary findings also suggest potential benefits in chronic neurological conditions, supporting its neuroprotective role within multimodal neurorehabilitation strategies. Standardized intervention protocols, mechanistic biomarkers, and longitudinal studies are required to strengthen clinical relevance and guide evidence-based integration into public health and lifestyle medicine.

## 1. Introduction

**Scheme 1:**
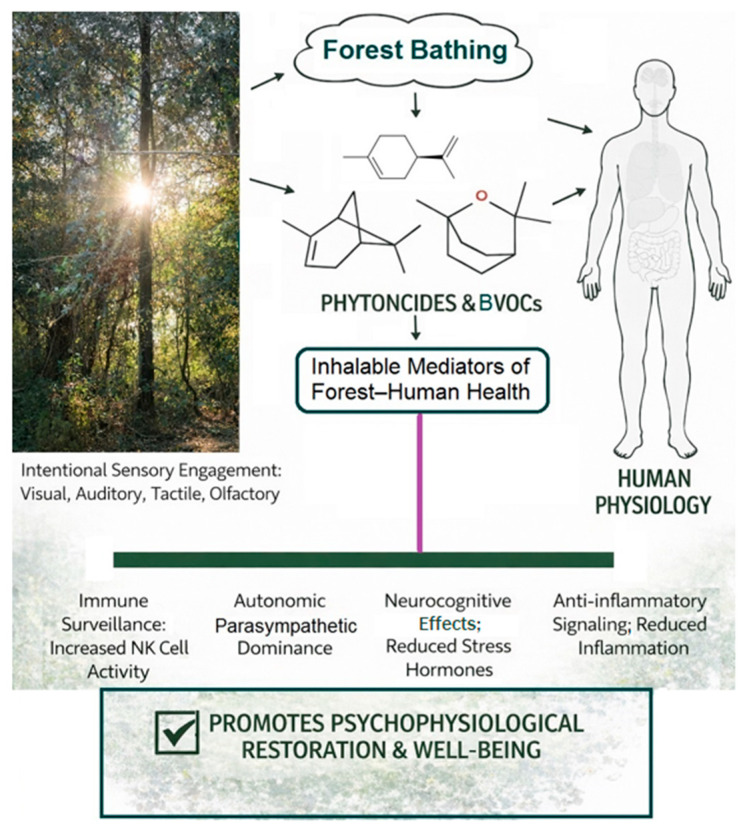
Schematic representation of the psychophysiological pathways of Forest Bathing (*Shinrin-yoku*) and the role of biogenic volatile organic compounds (*Green photograph courtesy of G.N. Roviello*).

The increasing global prevalence of stress-related disorders [1], immune dysregulation [2], and neurocognitive decline [3] has intensified the search for accessible, non-pharmacological strategies capable of supporting preventive maintenance. Modern lifestyles characterized by urbanization, environmental overstimulation, and reduced contact with natural environments [3] have contributed to physiological burdens that accumulate across the lifespan. Within this context, forest bathing, or *Shinrin-yoku* [4], has emerged as a structured nature-based practice with potential to mitigate some of these effects. More specifically, forest bathing refers to immersion in the forest atmosphere through multisensory engagement rather than physical exercise, as it involves simply being present in nature and connecting with it. Interestingly, in Japan, *Shinrin-yoku* is prescribed as a medical intervention in a manner similar to conventional treatments, with structured and repeated sessions administered over time [5]. Originally conceptualized as a public health initiative, it involves slow, mindful immersion in green environments with the aim of engaging the mentioned multiple sensory pathways and promoting psychophysiological restoration [4,6,7]. Forest bathing encourages attentive interaction with green surroundings and is distinct from general outdoor recreation because it emphasizes intentional sensory engagement, reduced cognitive load, and a contemplative pace. The forest environment provides a complex array of stimuli [8], including visual fractal patterns, natural sounds, organic scents, and biogenic volatile organic compounds [9,10] (BVOCs, Scheme 1).

These environmental elements are thought to elicit physiological responses through multiple pathways (Scheme 1), potentially mediated by bi-directional neuro-immune and metabolic–endocrine interactions, a mechanism well supported in the literature for these regulatory systems [11,12]. Remarkably, the clinical field study of Yu et al. suggested that forest exposure can improve cardiovascular function and psychological well-being, reducing heart rate, blood pressure, and negative mood while increasing positive emotion [13]. In parallel, emerging evidence from the same study, along with findings from the intervention study by Li et al., indicated that forest exposure can enhance cognitive restoration and recovery from fatigue, reflected in increased vigor, decreased fatigue, improved subjective sleep quality, reductions in tension, anger, depression, and confusion, and lower anxiety levels [13,14]. Previous studies have already addressed the relevance of forest bathing in preventing respiratory diseases and mitigating the impact of pandemics such as COVID-19 [15,16,17,18], as well as the necessity of protecting natural environments, including riparian [19,20] and coastal vegetation [21], to ensure that populations can benefit from the health-enhancing effects of nature, including the passive inhalation of tree-emitted volatile organic compounds. These contributions highlight the broader ecological and public health implications of maintaining biodiverse [22], intact natural landscapes capable of supporting human physiological resilience [22,23]. Despite growing interest, the scientific literature on forest bathing remains heterogeneous. Studies vary widely in exposure duration, environmental characterization, participant demographics, and biomarker selection [24]. Many investigations employ small sample sizes or short-term designs, limiting the ability to draw firm conclusions about long-term health impacts or underlying mechanisms [24]. Furthermore, the relative contributions of sensory, psychological, and biochemical components of forest environments remain insufficiently delineated [25]. These gaps highlight the need for a comprehensive synthesis that integrates findings across immune, endocrine, autonomic, and neurocognitive domains in relation to forest bathing [26]. In this context, the purpose of this narrative review is to examine forest bathing as a potential component of preventive medicine, with particular emphasis on aspects including immune modulation, stress physiology, and neuroprotective mechanisms [3]. By consolidating current evidence, including observed improvements in immune profiles, stress markers, and hematological parameters, alongside the identification of key variables like forest features and sensorial dimensions [27,28], this work aims to clarify the biological plausibility of forest-based interventions, identifying consistent patterns across studies, and ultimately highlighting methodological limitations that must be addressed to advance the field. In doing so, it seeks to provide a scientific foundation for the integration of forest bathing, supported by evidence of improved cardiovascular, metabolic, and antioxidant indexes and its recent recognition in clinical guidelines for hypertension [29,30], into broader preventive health strategies while outlining priorities for future research to strengthen clinical relevance.

### Methodology

To ensure a comprehensive overview of the current scientific landscape regarding forest bathing and its physiological impacts, a comprehensive literature search was conducted across multiple scientific databases, including Google Scholar, PubMed, Scopus, and Web of Science. The search strategy was performed using combinations of terms, including “forest bathing”, “*Shinrin-yoku*”, “forest therapy”, “nature-based interventions”, “forest medicine”, “immune modulation”, and “neuroprotection”. The search strategy focused on peer-reviewed articles exploring the intersection of factors such as forest-related practices and their links to immune modulation and psychophysiological processes with stress physiology, neuroprotective and neurocognitive outcomes. An initial search yielded 346 results. These records were subsequently screened based on specific inclusion criteria: studies published in English in peer-reviewed journals; research providing primary data or comprehensive syntheses on physiological, immunological, neuroprotective, or neurocognitive outcomes related to forest exposure; and studies involving both observational and interventional designs across diverse demographic groups. Additional books and theses were consulted where relevant to the thematic scope of this work. Following the removal of duplicates, retracted articles, and papers that were not peer-reviewed, 116 references were formally included in the bibliography. Although the full text cites 116 references, 30 are used to provide general context, while the core synthesis draws on more than 80 studies and further elaborates on selected literature found in the Introduction. Notably, a substantial portion of the literature cited in our work is predominantly recent; the papers published within the last five years represent about 70% of the total included references, revealing a surging contemporary interest in forest bathing for supporting immune regulation, stress reduction, neuroprotection, and neurocognitive resilience.

## 2. Biologic Responses and Physiological Outcomes of Forest Bathing

**Scheme 2:**
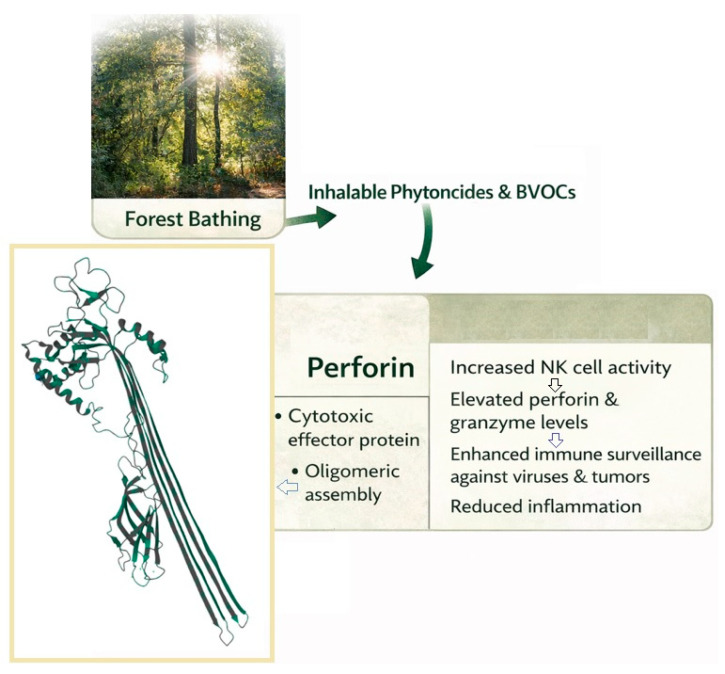
Schematic illustrating how forest bathing-derived inhalable compounds (phytoncides and BVOCs) may enhance immune surveillance. Exposure is associated with increased NK cell activity and elevated cytotoxic effector molecules, exemplified by perforin, shown here as a cryo-electron microscopy-derived three-dimensional structure (PDB ID: 7PAG [35]; https://www.rcsb.org/structure/7PAG (accessed on 9 January 2026)).

Human interaction with forest environments has historically been associated with feelings of relaxation and well-being, largely due to sensory factors such as visual greenery, natural sounds, clean air, and forest-derived aromas [15]. In Japan, this long-standing cultural practice was formally investigated through scientific research beginning in the early 2000s, leading to the emergence of Forest Medicine as a distinct interdisciplinary field [31]. Forest medicine draws upon concepts from environmental science, preventive medicine, and complementary health approaches to examine how forest exposure influences human physiological and psychological health [32]. Research in this area consistently indicates that forest bathing supports health maintenance not only by reducing stress and regulating autonomic nervous system activity, but also improving sleep quality, and enhancing emotional well-being. Together, these outcomes suggest that forest-based interventions may contribute meaningfully to the prevention of chronic, stress-related non-communicable diseases [29]. Mechanistically, a substantial body of evidence highlights immune modulation as a key biological effect of forest bathing [33]. Controlled observational studies involving short forest stays, and consequent exposure to BVOCs and phytoncides, have demonstrated increased activity and abundance of natural killer (NK) cells, alongside elevated levels of intracellular cytotoxic molecules, specifically effector proteins such as perforin (Scheme 2) and granzymes, which serve as a primary defense mechanism in immune surveillance against compromised or virally infected cells [34].

**Figure 1:**
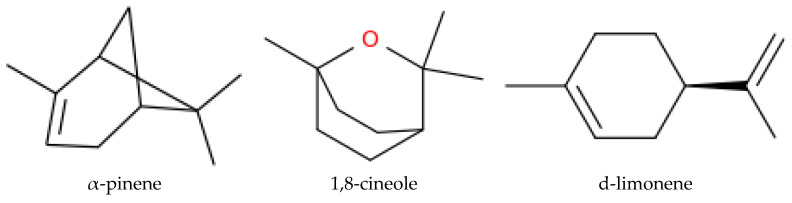
Chemical structures of the three purified phytoncides, α-pinene, 1,8-cineole, and d-limonene, examined alongside the complex plant stem oils reported in [41].

These immune enhancements are typically accompanied by reductions in stress-associated catecholamines and glucocorticoids, indicating a link between neuroendocrine regulation and immune function. Importantly, the persistence of elevated immune activity for several weeks following forest exposure suggests that even periodic engagement with forest environments may produce lasting biological effects [36,37]. Comparative studies further show that similar durations of urban exposure (e.g., walking in a city center or high-traffic areas) do not elicit these immune responses, revealing the distinct physiological influence of forest settings [38,39,40]. Growing mechanistic evidence suggests that phytoncides, including volatile monoterpenes and essential-oil constituents such as α-pinene, 1,8-cineole, d-limonene (Figure 1), and complex mixtures derived from *Chamaecyparis* (hinoki), *Thujopsis dolabrata* (hiba), and *Cryptomeria japonica* (sugi) stem oils, exert immunomodulatory activity by enhancing intracellular activation programs in human cytotoxic lymphocytes, thereby providing a plausible molecular basis for the health-supporting effects associated with forest environments [41].

Interestingly, experimental studies conducted in non-forest environments using controlled phytoncide exposure have reported immune activation patterns comparable to those observed during forest bathing, including enhanced immune activity and reduced stress hormone levels [37,42]. Beyond immunological outcomes, forest bathing has been associated with cardiovascular regulation, including lowered blood pressure, reduced heart rate, and improved heart rate variability. Psychological benefits, such as reduced anxiety, depression, fatigue, and improved mood and sleep quality, further reinforce the role of forest bathing as a preventive health intervention [29,43].

The randomized controlled field study of Ochiai et al. provides growing evidence that forest bathing exerts measurable preventive health effects through combined immunological, psychological, and stress-regulatory pathways [44]. Moreover, the randomized controlled crossover laboratory study by Longman et al. demonstrated that brief exposure to forest acoustic environments, compared to urban or industrial soundscapes, significantly enhances positive affect in adults, perceived restoration, and cognitive performance [45]. The immune changes following forest bathing sessions appear closely linked to concurrent shifts in mood and stress-related responses, supporting a psychoneuroimmunological mechanism underlying forest-based interventions [44,46]. Importantly, evidence suggests that even low-intensity and low-dose forest bathing sessions, such as two to three standardized exposures or short guided visits during work breaks, can yield benefits comparable to those observed in longer or more intensive interventions for mental well-being and stress resilience [47]. Real-world workplace studies further highlight the feasibility of integrating forest exposure into daily routines, with observed improvements in immune markers, and work-related attitudes among urban employees [47,48]. At the same time, conceptual analyses of the forest bathing literature emphasize the need to better characterize human–forest interactions, environmental features, and experiential conditions that shape health outcomes [48,49]. Together, these findings reinforce forest bathing as a practical, scalable preventive health strategy while demonstrating the importance of refined methodological frameworks to support clinical translation and evidence-based implementation [29,50,51]. To clarify the multidimensional nature of the above-mentioned health benefits, the table below provides a systematic overview of key studies investigating the physiological and psychological impacts of forest bathing, categorized by health domain, intervention characteristics, and primary outcomes (Table 1).

## 3. Integrated Systemic Regulation Underlying Forest-Based Health Effects

In this section we examine the physiological mechanisms through which forest environments exert systemic effects on human health and investigate how exposure to natural settings modulates immune function, attenuates inflammatory activity, and influences neuroendocrine and autonomic regulation. In particular, we focused on the multisensory and biochemical inputs characteristic of forest ecosystems, which have been shown to reduce stress reactivity, alter cytokine and hormonal profiles, and promote homeostatic stability. Together, these processes illustrate the capacity of forest environments to act as complex bioregulatory stimuli, contributing to improved physiological resilience and supporting both physical and psychological well-being.

### 3.1. Immunological Resilience and Inflammatory Regulation

Immersive exposure to natural forest environments has been shown to influence key physiological processes that maintain immune balance and contribute to lowering blood pressure [59]. Immersive exposure to low-latitude evergreen broad-leaved forests has been shown to improve sleep quality, mood, and immune function, supporting its role as a non-pharmacological intervention for holistic well-being [27]. In addition, compounds released by trees, such as the already mentioned phytoncides, may enhance the activity and cytotoxic function of CD8+ T and NKT cells, strengthening the body’s defenses against infections and abnormal cell growth [32,60]. Forest exposure also appears to modulate the expression of activating and inhibitory receptors on immune cells, supporting both immune activation and regulatory balance. Alongside these cellular effects, nature immersion can shift cytokine profiles toward an anti-inflammatory state, helping reduce systemic inflammation [61,62,63]. Overall, these interactions suggest that forest environments support immune resilience, promote inflammatory regulation, and offer a non-pharmacological strategy to improve overall health and prevent chronic disease [59,64]. The convergence of immunological modulation and neuroendocrine stabilization supports the concept of forest bathing as a systemic intervention operating through the psycho-neuro-endocrino-immune network, with implications for the regulation of physiological homeostasis [65]. Rather than merely suppressing or stimulating a single pathway, the multi-sensory and chemical input from the forest environment appears to facilitate a state of biological readiness, where the synchronization of cellular defenses and inflammatory control provides a foundation for the broader neurocognitive resilience currently being explored in clinical research [30]. This integrative perspective is further supported by emerging evidence from natural-product-based and biomolecular research, highlighting how bioactive compounds, peptides, and nature-inspired molecules can modulate immune defense, inflammatory signaling, metabolic pathways, and neurocognitive resilience across infectious, inflammatory, and neurodegenerative contexts [66,67,68,69,70,71,72,73].

### 3.2. Systemic Bioregulation: Autonomic Balance, Neuroendocrine Shifts, and Environmental Specificity

Field-based and review evidence consistently demonstrates that forest bathing is associated with measurable reductions in physiological stress and promotes autonomic nervous system regulation [74]. Controlled crossover experiments conducted across multiple forest sites in Taiwan and Japan show that short periods of walking or passive viewing in forest environments lead to lower salivary stress-related hormone levels, reduced blood pressure and pulse rate when compared with urban settings [13,53]. These physiological changes indicate a shift toward a relaxed and adaptive stress response, reflected by improved sympathovagal balance [75] and the previously mentioned harmonizing effects of natural environments across cardiovascular, endocrine, neural, and immune systems may play a particularly important role in stress-related outcomes [76,77]. Although methodological heterogeneity and small sample sizes limit causal inference, the convergence of evidence suggests that forest environments reduce allostatic load and enhance physiological resilience to stress [78]. In other words, these findings position *Shinrin-yoku* as a promising nature-based strategy for stress management, health promotion, and disease prevention within broader preventive-medicine and green public-health frameworks, while highlighting the need for larger, longitudinal, and methodologically robust studies [79,80,81]. Importantly, forest bathing has been increasingly studied for its ability to reduce stress and improve mental well-being [24], with research showing that spending time in forest environments can lower physiological markers of stress, including not only salivary but also serum cortisol, compared to urban settings, indicating a calming effect on the body’s stress-response system [52]. The intervention study of Li et al., which used a randomized crossover design, has also reported improvements in emotional well-being, with participants exhibiting reduced fatigue, better sleep quality, and increased positive feelings after forest exposure [14]. Biological changes, including elevated levels of serotonin, oxytocin, and insulin-like growth factor-1 [54], suggest that forest bathing may influence neuroendocrine pathways, contributing to both mental and physical health benefits [29]. Broader reviews on natural environments confirm that exposure to greenery generally supports stress reduction, in line with theories such as the Biophilia Hypothesis and Stress Recovery Theory [82,83,84], which posit an innate human affinity for life-sustaining landscapes and an evolved capacity for rapid physiological recovery when perceiving natural cues as safety signals [85]. These frameworks suggest that the restorative effects of the forest arise from evolved neural mechanisms that favor resource-rich environments, reducing modern stress responses through basic evolutionary tendencies [86]. Moreover, forest-based interventions have shown potential in managing chronic health conditions like hypertension in older adults [28]. This evidence has catalyzed an international shift toward formalizing forest-based health strategies, moving beyond individual well-being to address the “civilization diseases” associated with sedentary, urban lifestyles [87,88]. Comparative analyses of forest programs, particularly between Asian pioneers and European countries, demonstrate that integrating these natural assets into national preventive healthcare frameworks and social forestry dimensions can significantly improve the quality of life on a population level [28,89]. Beyond the generalized restorative effects of greenery, the clinical efficacy of forest immersion is increasingly viewed as a function of specific ecological characteristics that target physiological and psychological vulnerabilities [90]. Empirical data from diverse forest landscapes further suggests that these environments act as complex bioregulators, capable of stabilizing hemodynamic parameters and modulating the neurochemical substrates of affect. Seasonal variations and forest types appear to influence these effects significantly; for instance, visits to *Cinnamomum camphora* forests were found to reduce systolic and diastolic blood pressure and increase oxygen saturation especially in summer and autumn [56,91]. Similarly, different plant communities, such as mixed coniferous forests or mixed broad-leaved forests, exhibit distinct impacts on stress relief, emotional well-being, and physiological indicators like salivary stress-related hormones. These findings highlight the potential of thoughtfully designed urban green spaces and diverse plant communities to enhance public health, offering a scientific foundation for planning and optimizing natural environments for human well-being as further supported by the on-site experimental study of Zhang et al. [56]. The dynamic interplay between specific forest compositions and the resulting neuroendocrine shifts suggests that the “dose” of nature is not a generic variable, but one modulated by botanical diversity and seasonal bioactivity. This complexity seems to indicate that the body not only reacts to forest-derived sensory and biochemical inputs, but also undergoes integrated physiological shifts that may contribute to cognitive and neuroprotective outcomes.

## 4. Neurocognitive and Potential Neuroprotective Effects of Forest and Tree-Based Nature Exposure, with Associated Psychophysiological Responses

Forest environments are increasingly viewed as neurobiological systems that influence central nervous system function through integrated sensory, psychological, and biophysical inputs. Through multisensory cues forest bathing engages stress-responsive neural circuits, supports adaptive neuroplasticity, and may contribute to cognitive regulation by modulating prefrontal, limbic, and hippocampal networks involved in attention, emotional regulation, memory, and stress processing. The pragmatic controlled trial of McEwan et al. demonstrated that forest bathing improves psychological wellbeing and autonomic regulation, showing equivalence to an established wellbeing intervention [92]. Studies show that interacting with natural environments is associated with improvements in cognitive function, emotional regulation, memory, and sensorimotor processing, often mediated through region-specific changes in the brain, including stress-sensitive areas such as the amygdala and subiculum [93,94]. Additionally, exposure to natural environments has been shown to enhance heart rate variability and also reduce rumination, a key cognitive marker of psychological distress characterized by repetitive, self-referential negative thought patterns, indicating improved autonomic regulation and stress resilience [95]. Controlled trials, including the first pragmatic study in the United Kingdom, indicate that exposure to natural environments can provide wellbeing improvements comparable to established psychological interventions, with sustained effects on mood, nature connection, and physiological markers of neurocognitive health [95]. Systematic reviews further suggest that forests exert stronger neuroplastic effects than other types of green spaces, while residential greenness within proximal distances also offers measurable benefits [96]. These findings highlight the potential of forest and tree-mediated interventions to support adaptive neuroplasticity, mental health, and long-term brain resilience, suggesting the importance of integrating green spaces into public health strategies and sustainable urban design [92,97,98]. These integrated effects also align with emerging evidence suggesting potential neuroprotective benefits of forest and tree-based environments, as further outlined in Table 2 together with their cognitive implications.

### Cognitive and Neural Effects of Forest Environments

Recent research highlights the significant impact of forests on human cognitive function and brain health across the lifespan [99], with structural and functional neuroimaging studies identifying the prefrontal cortex, amygdala, and hippocampus as regions consistently influenced by environmental exposure and showing that greener, less urbanized settings are associated with enhanced structural integrity of these regions and reduced stress-related neural activity [100,101]. The controlled field study by Ramanpong et al. demonstrated that, in elderly populations, both structured and self-guided forest bathing significantly improved key cognitive domains, including attention, working memory, and creativity [26], as evidenced by performance gains in tasks such as the Stroop test, Forward Digit Span Task, and Remote Associates Test [102]. Notably, the benefits of forest bathing appear more strongly linked to the frequency of forest engagement rather than the total time spent or distance covered, suggesting that regular, repeated exposure is more critical than prolonged sessions [26,57,58]. The cognitive improvements are likely mediated by multiple mechanisms, including stress reduction, restoration of attentional resources (as proposed by Attention Restoration Theory), and multisensory stimulation that enhances cognitive flexibility and creative thinking [26,103]. Epidemiological studies further indicate that increased residential green space may slightly slow cognitive decline and support brain health, potentially through promoting physical activity, providing restorative environments, and reducing exposure to air pollution [104]. While some complex cognitive measures, such as backward digit span performance, may be less responsive in older adults, overall evidence reveals the potential of forest-based and green space interventions as accessible, low-cost strategies for maintaining cognitive function and promoting neurocognitive resilience in aging populations [26]. Complementing these findings, evidence from neuroimaging [93,94], environmental exposure studies [105], and mechanistic frameworks [106] indicates consistent modulation of stress-related neural circuits and associated cognitive benefits [107] (Table 2).

**Table 2:** Neurocognitive and potential neuroprotective effects of forest bathing and green space exposure.

Study/Focus	Intervention/Exposure	Key Cognitive/Neural Outcomes	Ref.
Environmental and Neurophysiological Studies	Urban vs. rural/green environments	Modulation of amygdala activity, hippocampal subfields, and physiological stress-recovery markers	[93,94]
Self-Guided and Structured Forest Bathing Programs	Multiple short forest visits or multi-session forest-healing programs over several weeks	Improvements in attention, working memory, creativity, and global cognitive function; enhanced memory, orientation, and emotional stability; frequency-dependent gains in attentional control	[26,108]
Forest Exposure and Alzheimer’s Disease and Related Dementias (ADRD) Risk	Green space exposure defined by duration, proximity, and frequency	Potential deceleration of cognitive decline. Preserved regional brain volumes and cortical thickness; modulation of neuroplasticity and inflammatory markers.	[96,99,105]
Theoretical and Mechanistic Studies	Forest bathing and green space exposure	Enhanced affective recovery, attentional restoration, and domain-specific cognitive resilience, supported by multisensory, stress-reduction, and neuroplasticity-related mechanisms	[82,83,84,106]
Forest walking or structured forest-therapy programs in middle-aged and older adults with elevated blood pressure	Forest walking or structured forest-therapy programs in middle-aged and older adults with elevated blood pressure	Improved mood, reduced anxiety, enhanced autonomic regulation, and significant blood-pressure reduction; psychophysiological pathways associated with lower neurocognitive decline and neurodegenerative risks	[13,29,53,56]
Chronic Neurological and Neurodegenerative Conditions (e.g., Stroke, Parkinson’s Disease, Dementia, Multiple Sclerosis, and age-related cognitive decline)	Forest therapy and nature-based rehabilitation programs; observational and interventional studies across neurological populations	Limited but suggestive benefits, including improvements in depressive and anxiety symptoms, functional recovery, autonomic regulation, quality of life, and domain-specific cognitive outcomes relevant to neurorehabilitation	[92,95,107]

In other terms, forests may act as complex bio-social environments capable of modulating neural circuits, immune pathways, and cognitive processes in concert. They are hypothesized to function as dynamic neuroecological systems, where psychological, sensory, and biophysical cues jointly influence neural plasticity and cognitive performance, with this integrative view raising the possibility that distinct forest attributes, ranging from tree canopy chemistry to spatial structure, could be mapped onto specific neurocognitive outcomes. Collectively, the findings of our work support the integration of forest-based therapies into preventive medicine frameworks, while also emphasizing the need for rigorously designed randomized controlled trials to strengthen causal inference and clinical translation [32,43,109,110].

## 5. Conclusions

*Shinrin-yoku*, or forest bathing, is a nature-based practice that supports preventive health by positively influencing the brain function, stress responses, and immune system [111]. Spending time in forests has been shown to increase immune activity, balance inflammatory signals, and lower stress hormones like cortisol, promoting immune resilience and reducing chronic inflammation [74]. It also enhances autonomic nervous system function, improving heart rate variability, lowering blood pressure, and fostering relaxation. Additionally, exposure to natural environments can support cognitive and emotional health, improving attention, memory, and creativity, likely through sensory engagement and stress reduction. These benefits highlight forest bathing as a practical, low-cost approach for maintaining physical and mental well-being, with potential applications in lifestyle medicine and public health initiatives [29]. Forest bathing represents a scientifically grounded, ecologically sustainable approach to preventive medicine, capable of influencing multiple physiological systems relevant to modern chronic disease burdens [31,112]. Regular immersion in forest environments may modulate immune pathways, a process closely linked to microglial-mediated immune surveillance in the brain [113], reduce systemic stress responses, and support neuroprotective pathways that are linked to cognitive resilience and emotional well-being [114]. Studies have examined the potential of forest therapy in chronic neurological conditions such as stroke (rehabilitation phase), Parkinson’s disease, dementia, and multiple sclerosis [107]; although evidence-based results are lacking, preliminary findings suggest possible benefits, supporting its proposed integration as a complementary component of multimodal neurorehabilitation [107]. Integrating forest-based practices into lifestyle medicine, urban planning, and community health programs may offer a practical strategy to counteract the health impacts of urbanization, sedentary behavior, and chronic stress [28,89]. As the field advances, interdisciplinary collaboration will be essential to fully elucidate the therapeutic potential of nature-based interventions and to position forest bathing as a validated component of preventive healthcare [22,115,116]. Importantly, forest bathing should be viewed not as a stand-alone therapeutic intervention, but as a complementary, low-risk strategy within lifestyle medicine and public health prevention frameworks. The convergence of immune, autonomic, and neurocognitive effects supports its biological plausibility as a means of enhancing physiological resilience in the context of chronic stress, urbanization, and aging populations. Future research priorities should include the development of standardized intervention protocols, improved environmental characterization of forest settings, and the use of robust mechanistic biomarkers across immune, endocrine, and neural systems. Additional efforts should also focus on the design and implementation of both healing gardens and indoor forest-bathing environments within public buildings such as schools, hospitals, churches and other places of worship, as well as other community facilities. Longitudinal and controlled studies on forest bathing will be essential to clarify dose–response relationships, durability of effects, and population-specific benefits. As interdisciplinary collaboration advances, forest bathing may become an evidence-informed component of preventive healthcare strategies that integrate ecological, physiological, and public-health perspectives.

## Figures and Tables

**Table 1:** Physiological and psychological effects of forest bathing.

Health Domain	Intervention Characteristics	Main Outcomes	Ref.
Immune modulation (innate immunity) and neuropsychological modulation	mindful forest walking vs. urban walking; forest acoustic exposure; two- vs. three-session forest therapy programs	Improvements in neuropsychological state (enhanced mood and vigor, reduced stress and negative affect); ↑ Salivary sIgA; ↓ cortisol; ↑ dopamine; improved mood/vigor and reduced stress/negative mood	[44,45,46]
Neuroendocrine stress regulation	Forest walking and viewing vs. urban exposure; forest-related neuroendocrine assessments	↓ Salivary cortisol; ↓ blood pressure; ↑ Serotonin, Oxytocin, and IGF-1;	[52,53,54]
Cardiovascular regulation	Forest bathing vs. urban exposure	↓ Blood pressure and heart rate; ↑ HRV; ↑ SpO2 (oxygen saturation)	[29,55,56]
Phytoncide-mediated immune effects	Indoor exposure to tree-derived essential oils	↑ NK cell activity; ↑ cytotoxic effector molecules; ↓ stress hormones	[37,41,42]
Dose–response and frequency effects of forest bathing	Comparison of session frequency (e.g., 2 vs. 3 sessions) and total duration	Comparable improvements in mental well-being across different session numbers; benefits evident even with low-dose or repeated short exposures	[26,47,57,58]
Immune surveillance (NK-cell activation)	Multi-day forest stays; periodic immersive engagement.	↑ NK cell activity and cytotoxic proteins (perforin, granulysin, granzymes); effects lasting up to 30 days	[34,36,37]

## Data Availability

No data available from this literature review.

## Abbreviations

The following abbreviations are used in this manuscript:

ADRD	Alzheimer’s Disease and Related Dementias
ANS	Autonomic Nervous System
BVOCs	Biogenic Volatile Organic Compounds
CD8+ T	Cytotoxic T cells
DMN	Default Mode Network
FDST	Forward Digit Span Task
HPA	Hypothalamic–Pituitary–Adrenal
HRV	Heart Rate Variability
IGF-1	Insulin-like Growth Factor-1
NK	Natural Killer
NKT	Natural Killer T
RAT	Remote Associates Test
sgPFC	Subgenual Prefrontal Cortex
sIgA	Salivary Immunoglobulin A
SRT	Stress Recovery Theory
VOCs	Volatile Organic Compounds
